# Evaluating the Use of Large Language Models in Improving the Readability of Online Patient Education Materials for Peripheral Nerve Surgery

**DOI:** 10.3390/healthcare14121640

**Published:** 2026-06-10

**Authors:** Nikhil Sriram, Rishi Jain, Mehul Mittal, Ravi A. Patel, Benjamin J. Fredeen, Anitesh Bajaj, Rebecca Du, Karl Habashy, Hanna Kemeny, Sachin Govind, Kevin Swong, Christopher S. Ahuja, Najib El Tecle

**Affiliations:** Department of Neurological Surgery, Northwestern University Feinberg School of Medicine, Chicago, IL 60611, USA

**Keywords:** artificial intelligence, ChatGPT, Gemini, large language models, neurosurgical education, patient education

## Abstract

**Objective:** Online patient education materials (OPEMs) are important resources for patients seeking health information. While the National Institutes of Health (NIH) and American Medical Association (AMA) recommend a sixth-grade readability level for OPEMs, commonly available material often exceeds such criteria. Large language models (LLMs), such as ChatGPT and Gemini, have emerged as tools for health education with potential applications in simplification of health material. This study assesses the utility of ChatGPT and Gemini in enhancing the readability of OPEMs for peripheral nerve surgeries. **Methods:** Eleven common peripheral nerve surgeries were used as online search terms. The first 20 unique search results were assessed; results were excluded if they did not include patient-facing material. ChatGPT and Gemini were instructed to rewrite the text of the OPEM at or below a sixth-grade reading level. Readability metrics were calculated for original OPEMs, alongside ChatGPT and Gemini rewrites. LLM responses were reviewed for accuracy/quality (five-point scale) and comprehensiveness (three-point scale) using predefined criteria. **Results:** A total of 220 websites were assessed. In total, 155 OPEMs met the inclusion criteria; 65 websites were excluded because they were academic journal articles or other provider-facing materials. The average Flesch–Kincaid grade level (FKGL) of OPEMs was 11.3, significantly greater than the NIH/AMA-sixth grade recommendations (*p* < 0.001). The average FKGL of ChatGPT rewrites was significantly lower than that of OPEMs (11.3 vs. 7.5, *p* < 0.001), as was the average FKGL of Gemini rewrites (11.3 vs. 5.6, *p* < 0.001). ChatGPT rewrites were of higher accuracy/quality (4.5/5.0 vs. 4.0/5.0, *p* < 0.001) and comprehensiveness (2.0/3.0 vs. 1.0/3.0, *p* < 0.001) relative to Gemini rewrites. **Conclusions:** The readability of online patient education materials for peripheral nerve surgery significantly exceeded NIH/AMA recommendations. ChatGPT and Gemini were able to significantly simplify the reading level of these OPEMs. LLMs may serve as tools to improve the readability of peripheral nerve surgery OPEMs.

## 1. Introduction

Online patient education materials (OPEMs) are important resources for health education, providing details about conditions and various treatment modalities to inform patients. Despite their importance, studies demonstrate that these resources are often written at reading levels above the sixth-grade standard recommended by the National Institutes of Health (NIH) and American Medical Association (AMA) [1,2,3]. The field of neurosurgery is no exception; prior studies have consistently revealed a disconnect between the level of language used in patient-facing materials and general public health literacy across several subspecialties [4,5,6]. These deficits may leave patients misinformed about the benefits and risks of procedures, treatment optionality, and coverage of care.

Advances in artificial intelligence (AI) have offered potential solutions to these challenges, such as simplifying the academic literature for patients. In a recent study, Guerra and colleagues highlighted significant reductions in validated reading grades by utilizing large language models (LLMs) to rewrite academic neurosurgical text [7]. Prior studies have extended these methods directly to OPEMs for a range of medical specialties, including within neurosurgery, with notable success in improving the readability of materials [8,9,10]. As publicly available LLMs such as OpenAI’s ChatGPT and Google Gemini become increasingly adaptive and capable of complex reasoning, their performance on such tasks shows continued improvement.

As the number of individuals in the United States affected by peripheral neuropathies continues to grow beyond an estimated 20 million [11], OPEMs are a critical resource in the space, providing patients with adequate information to make informed decisions. Prior analysis has shown that OPEMs for peripheral nerve surgery, including carpal tunnel surgery, nerve repair, nerve grafting, and various other hand surgeries, have readability levels exceeding the aforementioned recommendations [12,13,14,15,16,17]. The literature has begun to explore the utilization of artificial intelligence within peripheral nerve surgery-related education, with a focus on carpal tunnel release [18,19,20,21,22,23]. The present study expands on the prior literature by including several additional peripheral nerve surgical procedures and compares two LLMs to capture differences in performance. In this study, we assess the performance of ChatGPT and Gemini in improving the readability of online patient education materials for peripheral nerve surgery. We hypothesize that ChatGPT and Gemini will effectively improve the readability of online patient education materials for peripheral nerve surgery to align with recommended reading levels.

## 2. Methods

### 2.1. Website Collection

To collect OPEMs for readability analysis, a Google search was conducted for relevant websites. The following search terms were queried, representing several common peripheral nerve surgical procedures: “carpal tunnel release,” “ulnar nerve decompression,” “brachial plexus surgery,” “nerve graft surgery,” “nerve transfer surgery,” “thoracic outlet decompression,” “nerve entrapment surgery,” “Morton’s neuroma surgery,” “tarsal tunnel release,” “peripheral nerve tumor surgery,” and “peripheral nerve stimulation.”

To minimize bias in search results, searches were performed in Incognito mode, with browser and computer location turned off, browser data cleared, and cache disabled. All website searches were performed between 12 and 18 January 2025. The first 20 unique search results for each search term were assessed. Results were excluded if they did not include free, publicly available patient-facing material. These included academic journal articles and other provider-facing materials such as continuing medical education materials, clinical practice guidelines, product advertisements, surgical technique education videos, or operative technique manuals. OPEMs were categorized into one of three website types: academic/hospital (e.g., Cleveland Clinic), private practice, or online health reference (e.g., WebMD). Classifications of this nature have been established in several prior studies [24,25,26,27]. To ensure uniform categorization, OPEMs with ambiguous categorization were reviewed jointly with another study team member to reach consensus classification. Other characteristics, including links to health care services, discussion of insurance coverage, and inclusion of educational photos or videos, were also recorded for each website.

### 2.2. LLM Data Collection

Once all websites had been collected, analyses using two publicly available LLMs were conducted: ChatGPT 4.0 (OpenAI, San Francisco, CA, USA) and Gemini 1.5 Flash (Google, Mountain View, CA, USA). These models were selected because they are widely available, free to use, and popular amongst the public. The free text from each OPEM was copied into each LLM with the following prompt to produce a simplified rewrite: “Rewrite the above patient education material at or below a sixth-grade reading level”. Similar bias mitigation techniques were employed for LLM analysis as previously described for website collection, such as queries performed in Incognito mode with location and cache disabled. In addition, LLM memory was turned off between successive queries. No adjustments were made to LLM temperature settings or other generation parameters in ChatGPT or Gemini—default settings were used. All LLM rewrites were generated between 19 and 25 January 2025.

### 2.3. Readability Analysis

Following the collection of websites and LLM rewrites, a readability analysis was performed. Automated analysis was conducted using WebFX [28]. WebFX contains several readability metrics, including grade-level indicators and statistics on the number of sentences, words, and complex words, all of which can be used to study the complexity of text. For this study, the following metrics were utilized: Flesch–Kincaid grade level (FKGL), Flesch–Kincaid reading ease score (FKRE), Gunning fog score, simple measure of gobbledygook (SMOG) index, Coleman–Liau Index, and automated readability index. These readability measures are commonly utilized, validated metrics for health literacy research [2,3,4,5,6,7,8,9,10,12]. The free text of each website was passed through to collect the readability metrics. Subsequently, the free text of each LLM response was passed through to collect the readability metrics for the corresponding rewrite.

### 2.4. Accuracy and Comprehensiveness Analysis

To evaluate the accuracy/quality and comprehensiveness of the LLM-generated content, two neurosurgery residents (R.D., K.H.) independently evaluated 25% of the ChatGPT and Gemini rewrites. The evaluated rewrites were randomly selected using a random number generator. For each number randomly generated, the corresponding ChatGPT and Gemini rewrites were selected for review. Reviewers were blinded regarding which LLM generated which rewrite. The methodology followed in this study aligned with methodologies described in prior studies [7,29].

The two reviewers scored the LLM-generated rewrites on two domains: accuracy/quality (1–5 scale) and comprehensiveness (1–3 scale). Reviewers were trained on the domain criteria by performing supervised scoring prior to independent evaluation to ensure consistent application of scoring criteria. The definitions of the scoring scale values are provided in Table 1. All scoring was completed relative to the original OPEM source, as judged by the reviewer. Scores by the reviewers were averaged to provide composite accuracy/quality and comprehensiveness scores.

### 2.5. Statistical Analysis

Descriptive statistics were calculated for the various quantitative readability and quality metrics for both OPEM and LLM responses and reported as means with standard deviations. Readability metrics were compared between OPEMs based on website type or search term using one-way ANOVA followed by the Tukey honestly significant difference (HSD) post hoc test. A multivariate linear regression model was used to assess the association between website characteristics (links to healthcare services, discussion of insurance coverage, presence of educational photos, and presence of educational videos) and OPEM Flesch–Kincaid grade level.

Readability metrics between OPEMs, ChatGPT rewrites, and Gemini rewrites were compared using a repeated-measures ANOVA to account for the within-subjects (paired) design. When ANOVA tests were significant, pairwise comparisons were conducted using estimated marginal means with Tukey adjustment for multiple comparisons. Normality assumptions were assessed using Shapiro–Wilk tests, supplemented by visual inspection of Q–Q plots. Where deviations from normality were observed, nonparametric Friedman tests were conducted as sensitivity analyses. When Friedman tests were significant, post hoc pairwise comparisons were conducted using Dunn’s test with Bonferroni adjustment. Accuracy/quality and comprehensiveness data were treated as ordinal data and were reported as medians with interquartile ranges (IQRs); statistical comparisons between LLMs utilized Wilcoxon signed-rank tests. A one-sample *t*-test was used to compare the FKGL of OPEMs, ChatGPT rewrites, and Gemini rewrites respectively to the NIH/AMA sixth-grade recommendations. The significance level for all statistical tests was set at <0.05. All statistical analyses were performed in R (4.3.1).

## 3. Results

**Figure 1:**
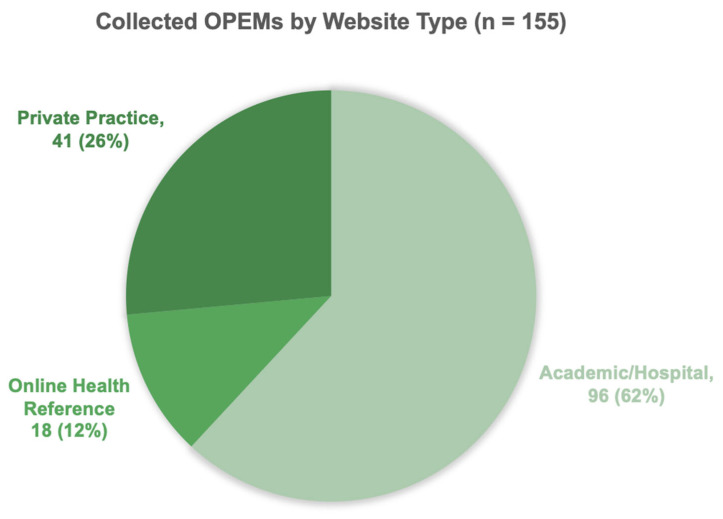
Aggregated OPEMs by website type.

A total of 220 websites were analyzed across the 11 search terms. In total, 155 websites met the inclusion criteria and were included in subsequent analysis. Of the 65 excluded websites, 44 (67.7%) were academic journal articles, nine (13.8%) were continuing medical education articles, five (7.7%) were clinical practice guidelines, three (4.6%) were product advertisements, two (3.1%) were surgical technique education videos, and two (3.1%) were operative technique manuals. Thus, a total of 155 ChatGPT rewrites and 155 Gemini rewrites were generated. Of these, 96 (61.9%) were academic/hospital websites, 18 (11.6%) were online health reference sites, and 41 (26.5%) were private practice websites (Figure 1).

**Figure 2:**
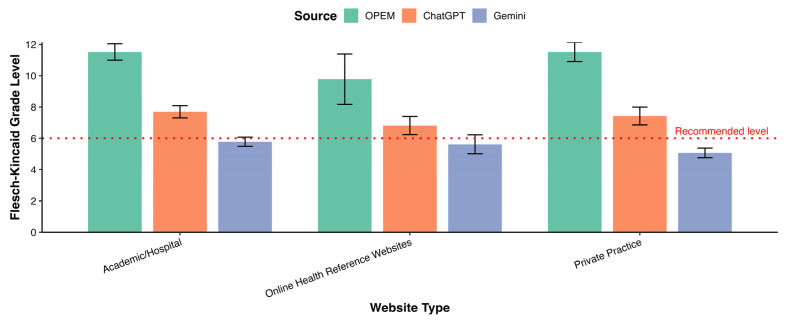
Flesch–Kincaid grade level by website type.

**Figure 3:**
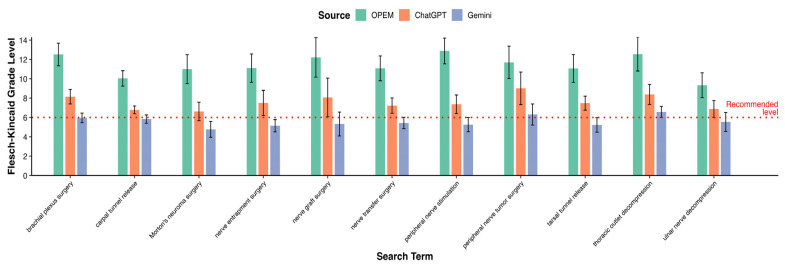
Flesch–Kincaid grade level by search term.

The average FKGL of OPEMs was 11.3. Online health reference websites had a significantly lower FKGL compared to academic/hospital websites (9.8 ± 3.2 vs. 11.5 ± 2.6, *p* = 0.022) and private practice websites (9.8 ± 3.2 vs. 11.5 ± 1.9, *p* = 0.042) (Figure 2). The search term “peripheral nerve stimulation” had the highest FKGL of 12.9, while the search term “ulnar nerve decompression” had the lowest FKGL of 9.3 (Figure 3). In total, 122 (78.7%) OPEMs included direct links to healthcare services or providers, 22 (14%) included a discussion of insurance coverage for procedures, 62 (40.0%) included educational photos about the procedure, and 36 (23.2%) included educational videos about the procedure. On multivariate analysis, the presence of direct links to healthcare services or providers was associated with significantly higher FKGL (β = 1.08, *p* = 0.034) after adjustment. Inclusion of educational photos was associated with significantly lower FKGL (β = −1.24, *p* = 0.003) after adjustment. Discussion of insurance coverage and inclusion of videos were not significantly associated with FKGL after adjustment.

Compared to the FKGL of OPEMs, the average FKGL of ChatGPT rewrites was significantly lower (11.3 ± 2.6 vs. 7.5 ± 1.8, *p* < 0.001), as was the FKGL of Gemini rewrites (11.3 ± 2.6 vs. 5.6 ± 1.3, *p* < 0.001). The FKGL of Gemini rewrites was significantly lower than that of ChatGPT rewrites (5.6 ± 1.3 vs. 7.5 ± 1.8, *p* < 0.001). OPEMs had an average FKRE score of 48, considered “difficult” to read. ChatGPT rewrites had an average FKRE score of 66.7, considered a “standard” level for reading. Gemini rewrites had an average FKRE score of 76.0, interpreted as “fairly easy” to read [30,31]. Full results across all readability metrics are shown in Table 2.

Throughout the six readability metrics studied—FKGL, FKRE, Gunning fog score, SMOG index, Coleman–Liau Index, and automated readability index—there was a significant difference between OPEMs and ChatGPT, OPEMs and Gemini, and ChatGPT and Gemini (*p* < 0.001) (Table 2). Nonparametric sensitivity analyses using Friedman tests with Dunn test post hoc comparisons yielded results consistent with those of the repeated-measures ANOVA. The FKGL of Gemini rewrites (5.6 ± 1.3) was on par with the NIH/AMA sixth-grade recommendations, while the FKGL of OPEMs (11.3 ± 2.6) and ChatGPT rewrites (7.5 ± 1.8) were both significantly greater than NIH/AMA sixth-grade recommendations (Figure 2 and Figure 3). ChatGPT and Gemini rewrites also showed lower variance in response readability level (Table 2).

When gauging performance across website types, the average FKGL for ChatGPT rewrites was 7.7 for academic/hospital website revisions, 6.8 for online health reference website revisions, and 7.4 for private practice website revisions; no significant differences were observed (Figure 2). For Gemini rewrites, the average FKGL was 5.8 for academic/hospital website revisions, 5.6 for online health reference website revisions, and 5.1 for private practice website revisions, all below the sixth-grade recommendations from the NIH/AMA (Figure 2). Gemini rewrites of private practice websites were of significantly lower FKGL compared to Gemini rewrites of academic/hospital websites (*p* = 0.011).

Across search terms, the average FKGL for ChatGPT rewrites was lowest for Morton’s neuroma surgery (6.6) and highest for peripheral nerve tumor surgery (9.0) (Figure 3). The average FKGL for Gemini rewrites was lowest for Morton’s neuroma surgery (4.8) and highest for thoracic outlet decompression (6.6) (Figure 3).

The ChatGPT rewrites had a median quality/accuracy rating of 4.5/5.0, with 85% of responses scored 4+, interpreted as high quality with minimal to no inaccuracies. In comparison, the Gemini rewrites had a median quality/accuracy rating of 4.0/5.0, with 60% of responses scored 4+ (Table 3). The reviewers scored 58% of ChatGPT rewrites as having equivalent comprehensiveness to the original OPEM; the median comprehensiveness score was 2.0/3.0. In comparison, 5% of Gemini rewrites were rated as having equivalent comprehensiveness to the original OPEM; the median comprehensiveness score was 1.0/3.0 (Table 3).

## 4. Discussion

Peripheral nerve surgeries, which are common and frequently elective outpatient surgical procedures, are of increasing interest for patients, clinicians, and researchers alike [12,32,33]. As patients seek autonomy within the care process and a deeper understanding of the treatment they are receiving, OPEMs serve as important sources; however, these materials are consistently written above NIH/AMA-recommended reading levels. LLMs, such as ChatGPT and Gemini, have shown potential in improving readability of OPEMs across several specialties, including within neurosurgery [7,8,9,10,21,22,23]. This study aimed to evaluate the effectiveness of ChatGPT and Gemini in improving the readability of OPEMs related to peripheral nerve surgery.

Across assessed text conversions, ChatGPT and Gemini demonstrated superior readability metrics relative to the original OPEM material. Between the two models, Gemini rewrites had superior readability across all metrics, and Gemini was most consistently in line with the sixth-grade reading level recommendation from the NIH/AMA. Both ChatGPT and Gemini rewrites showed reduced variance in readability level compared to the original OPEMs and generally showed similar levels of simplification across various website types and search terms.

The average neurosurgical OPEM has an FKGL of approximately 10th grade [34]. Analyses within subspecialities of neurosurgery have shown similar results [4,5,6]. In line with the results of our study, prior studies have found that OPEMs for various peripheral nerve surgeries consistently exceeded the NIH/AMA-recommended sixth-grade reading level [12,13,14,15,16,17]. The prior literature has also shown that neurosurgical patients commonly have poor recall of information following a visit and often leave with their care goals unmet, in part due to the complexity of provided information [35]. As such, the use of LLMs may serve as a valuable tool to improve material readability in neurosurgery.

Critically, we highlight that not all tools may possess the same strengths; in our analyses, Gemini more consistently improved the readability of material. In a study assessing the readability of radiology-related patient education materials, Gupta et al. similarly found a lower reading level among Gemini text rewrites than for ChatGPT [36]. However, additional analyses by neurosurgery resident reviewers in our study indicated that ChatGPT-generated materials were of higher quality/accuracy and comprehensiveness than Gemini-generated materials. A greater number of ChatGPT rewrites were scored as high quality with minimal to no inaccuracies. A lower percentage of Gemini rewrites maintained the same level of comprehensiveness relative to the original OPEM. This aligns with the prior literature noting that ChatGPT rewrites retain a greater degree of information and tend to have more appropriate and clear responses relative to Gemini rewrites [36]. The differences noted between the two models in this study and the prior literature may potentially reflect inherent model architecture differences that favor certain syntax structures or conversation format, alongside potential variance in training data and default parameter settings. However, additional investigations across specialties are needed with evaluation of larger numbers of LLM responses to better characterize differences in accuracy/quality and comprehensiveness between models and the reasons for these differences; these investigations should also include variations in prompting to further understand model performance. The inverse relationship between improved readability and worsened quality/accuracy is an important observation that has also been noted for OPEMs in the prior literature [24,37]. Improved readability of material using LLMs may similarly sacrifice comprehensiveness and/or quality. Additional studies assessing strategies to limit content loss from LLM rewrites improved readability, potentially utilizing novel training paradigms or specialized prompting, are necessary to further investigate this question.

Our findings, in parallel with findings of prior studies, indicate that OPEMs require revision to improve information readability for patients undergoing peripheral nerve surgeries. LLMs can serve as an effective and easy-to-use conduit for converting information into a form that aligns with reading level recommendations. However, it is important to acknowledge that LLMs are subject to hallucinations or factual errors, which can manifest as misrepresentation of information or presentation of fabricated or false information, which may mislead a reader [38,39]. This is reflected in our study, as not all LLM rewrites achieved the highest possible accuracy/quality scores. Thus, caution remains necessary when interpreting LLM output. Further research is also needed to better delineate the differences in performance between the two models; however, the results of this study suggest that both are effective in improving readability of OPEMs for peripheral nerve surgery.

The present study is not without limitations. As LLMs continue to evolve, this research represents an early but static, cross-sectional snapshot of the state of AI in peripheral nerve surgery OPEMs. In addition, the websites surveyed in this study may be updated periodically and could generate different readability scores in the future. Analysis of the first 20 Google results for each search term may reflect the influence of institutional prominence, search engine optimization practices, and geographic indexing, which may limit generalizability. Additionally, we note that readability metrics serve as proxies for patient comprehension, rather than direct measures of it. Thus, it is important to acknowledge that whether simplified LLM-generated texts for peripheral nerve surgery OPEMs improve patient understanding, recall, or decision-making remains untested and should be a focus of future work. In addition, depending on patients’ degree of health literacy, a single readability target, such as a sixth-grade reading level, may not be appropriate for all patient populations. Future studies should aim to investigate differences in patient preferences and comprehension between OPEMs and LLM-generated material. In addition, ChatGPT and Gemini are constantly being trained on the information they encounter and process—readability results may differ based on the sequence and date of prompts and may continue to evolve as new models are released. Ongoing updates and variability across sessions can also result in differences in generated output, even with standardized prompting and browser controls. Differences in prompting structure and phrasing may also influence the reading level that models generate output. This study utilized a single prompt, and the results reflect how the two included models responded to that specific prompt. Future studies should study the impact of variations in prompt phrasing on the reading level of LLM-generated education materials. Further analysis of accuracy/quality and comprehensiveness on a diverse array of LLM-generated materials is needed to better characterize the differences between ChatGPT and Gemini. Moreover, other publicly available LLMs, such as DeepSeek or Claude, have also been studied as patient education tools in other fields and should be further explored within peripheral nerve surgery [40,41]. Nonetheless, this study offers a thorough investigation of LLM simplification of patient education material for peripheral nerve surgery, with both ChatGPT and Gemini meaningfully improving the readability of online materials.

## 5. Conclusions

The findings of this study demonstrate that the readability of OPEMs for peripheral nerve surgery significantly exceeds recommended reading levels. Both ChatGPT and Gemini are effective in improving the readability of OPEMs for peripheral nerve surgery. Gemini-generated content simplified OPEMs to a greater degree, while ChatGPT-generated information was generally rated as higher quality and more comprehensive. LLMs may be used as tools to improve the readability of OPEMs for peripheral nerve surgery.

## Figures and Tables

**Table 1:** Definition of quality/accuracy score and comprehensiveness score.

Metric	Scoring Definition
Quality/accuracy score (1–5)	1: Very poor quality and/or majority inaccurate information relative to original source; 2: Poor quality and/or substantial inaccurate information relative to original source; 3: Moderate quality with some inaccurate information relative to original source; 4: High quality with minimal inaccurate information relative to original source; 5: Maintained complete accuracy relative to original source (little to no difference in information between original source and LLM rewrite).
Comprehensiveness score (1–3)	1: LLM response is less comprehensive than the original source; 2: LLM response is equal in comprehensiveness to the original source; 3: LLM response is more comprehensive than the original source.

**Table 2:** Readability metrics across OPEMs, ChatGPT-generated rewrites, and Gemini-generated rewrites.

Readability Metric	Online Patient Education Materials (OPEMs)	ChatGPT Rewrites	Gemini Rewrites	OPEM- ChatGPT *p*-Value	OPEM- Gemini *p*-Value	ChatGPT- Gemini *p*-Value
Flesch–Kincaid reading grade level	11.3 [2.6]	7.5 [1.8]	5.6 [1.3]	<0.001	<0.001	<0.001
Flesch–Kincaid reading ease score	48.0 [13.0]	66.7 [8.2]	76.0 [7.1]	<0.001	<0.001	<0.001
Gunning fog score	14.0 [2.6]	9.4 [1.8]	7.7 [1.3]	<0.001	<0.001	<0.001
SMOG index	10.5 [2.0]	7.4 [1.3]	6.2 [1.0]	<0.001	<0.001	<0.001
Coleman–Liau index	13.7 [2.4]	11.9 [1.4]	10.5 [1.4]	<0.001	<0.001	<0.001
Automated readability index	11.9 [3.1]	8.0 [2.3]	5.5 [1.7]	<0.001	<0.001	<0.001

Results reported as mean [SD]. Repeated-measures ANOVA followed by Tukey post hoc test performed for each metric. Tukey results are presented in OPEM-ChatGPT *p*-value, OPEM-Gemini *p*-value, and ChatGPT-Gemini *p*-value columns. Higher Flesch–Kincaid reading ease score indicates higher level of readability; lower score for all other metrics indicates higher level of readability. Abbreviations: SMOG = simple measure of gobbledygook.

**Table 3:** Quality/accuracy and comprehensiveness scores of ChatGPT and Gemini rewrites.

	ChatGPT	Gemini	*p*-Value
Quality/accuracy score (1–5)	4.5 [4.5–5.0]	4.0 [3.5–4.5]	<0.001
Comprehensiveness score (1–3)	2.0 [2.0–2.0]	1.0 [1.0–1.5]	<0.001

Results reported as median [interquartile range]. *p*-values generated using Wilcoxon signed-rank tests.

## Data Availability

The data presented in this study are openly available in FigShare at https://doi.org/10.6084/m9.figshare.32068944.
